# Field Tests for Evaluating the Aerobic Work Capacity of Firefighters

**DOI:** 10.1371/journal.pone.0068047

**Published:** 2013-07-02

**Authors:** Ann-Sofie Lindberg, Juha Oksa, Désirée Gavhed, Christer Malm

**Affiliations:** 1 Sports Medicine Unit, Umeå University, Sweden; 2 Winternet, Boden, Sweden; 3 Physical Work Capacity-team, Finnish Institute of Occupational Health, Oulu, Finland; 4 Department of Women’s and Children’s Health, Karolinska Institute, Sweden; University of Granada, Spain

## Abstract

Working as a firefighter is physically strenuous, and a high level of physical fitness increases a firefighter’s ability to cope with the physical stress of their profession. Direct measurements of aerobic capacity, however, are often complicated, time consuming, and expensive. The first aim of the present study was to evaluate the correlations between direct (laboratory) and indirect (field) aerobic capacity tests with common and physically demanding firefighting tasks. The second aim was to give recommendations as to which field tests may be the most useful for evaluating firefighters’ aerobic work capacity. A total of 38 subjects (26 men and 12 women) were included. Two aerobic capacity tests, six field tests, and seven firefighting tasks were performed. Lactate threshold and onset of blood lactate accumulation were found to be correlated to the performance of one work task (r_s_ = −0.65 and −0.63, p<0.01, respectively). Absolute (mL·min^−1^) and relative (mL·kg^−1^·min^−1^) maximal aerobic capacity was correlated to all but one of the work tasks (r_s_ = −0.79 to 0.55 and −0.74 to 0.47, p<0.01, respectively). Aerobic capacity is important for firefighters’ work performance, and we have concluded that the time to row 500 m, the time to run 3000 m relative to body weight (s·kg^−1^), and the percent of maximal heart rate achieved during treadmill walking are the most valid field tests for evaluating a firefighter’s aerobic work capacity.

## Introduction

Working as a firefighter is physically strenuous, and rescue during smoke diving with breathing apparatus (BA) is considered the most demanding work performed by firefighters [1], [2], [3], [4], [5]. The metabolic demands for firefighters’ work performance, expressed as relative oxygen consumption (VO_2_ in mL·kg^−1^·min^−1^), range between 16 and 55 mL·kg^−1^·min^−1^. The wide range in metabolic demands most likely depends on the pace and type of work task investigated. Consequently, the results reflect the linear correlation between submaximal workload and oxygen consumption [6], [7]. Metabolic demands are also affected by increased body temperature [8], the use of personal protective gear [9], [10], [11], [12], and emotional stress [13].

In addition to competency in firefighting skills, a high level of physical fitness in terms of aerobic capacity, anaerobic capacity, muscular strength, and endurance prevents injuries and increases the firefighter’s ability to cope with the overall physical stress they face in their profession [14], [15]. Determination of maximal aerobic capacity (VO_2max_) among firefighters has been performed with both direct measurement of VO_2max_ and indirect estimations. Results vary depending on the test mode (running, biking, etc.) with a mean range of 39.6–61.0 mL·kg^−1^·min^−1^ [16]. A minimum relative VO_2max_ of 39–45 mL·kg^−1^·min^−1^ [12], [17], [18], [19], [20] and absolute VO_2max_ of 2.7–4.0 L·min^−1^ [12], [21] has been proposed for firefighters. Direct measurement of VO_2max_, however, is complicated, time consuming, and expensive and such tests are, therefore, less than optimal as a standard procedure within rescue services. It may be more efficient and feasible to test firefighters using indirect estimations of VO_2max_ with the assumption that such indirect tests may serve equally well for prediction of physical work performance. Maximal anaerobic capacity among firefighters is rarely investigated [16], [22], [23], [24] and, in contrast to aerobic capacity, no minimum limits have ever been suggested.

In Sweden, a 6 min walking test on a treadmill at 4.5 km·h^−1^ and an 8° incline has to be completed for entry into rescue service education and for permission to execute smoke diving in accordance with government regulations [25]. Additional physical testing, not governed by regulations, is also carried out by individual municipalities. These physical tests are not based on scientific studies, nor are they standardized, and they are thus open for biased selection of firefighters. It is important that results from selected physical tests correlate with the true work capacity of firefighters to avoid unreasonable discrimination due to non-relevant, confounding factors such as gender differences.

The first aim of the present study was to evaluate the correlations between direct (laboratory) and indirect (field) aerobic capacity tests with commonly occurring, and physically demanding, firefighting tasks. The second aim was to give recommendations as to which field tests may be useful for evaluating firefighters’ aerobic work capacity. Both aims are achieved and useful field tests are recommended.

## Methods

### Subjects

After receiving written and verbal explanations of the procedure, 42 subjects volunteered to participate and 38 subjects completed the study. Subjects included male full-time firefighters (MFF, n = 8), male part-time firefighters (MPF, n = 10), and civilian men (CM, n = 8) and women (CW, n = 12) with no experience working as a firefighter. The mean ± Standard deviation (SD) (min and max) age, weight, body mass index (BMI), and B-HB for the 38 subjects completing the study were 34±9.8 (20–57) years, 78±11.1 (53–107) kg, 25±2.7 (20–32) kg·m^2^, and 149±12.7 (105–174) g·L^−1^, respectively, and no significant differences were observed between subject groups. The CW group was shorter than the MPF and CM (Table 1). No female firefighters were available to participate in this study.

**Table 1:** Anthropometrics and physical tests performance.

Profession		Firefighters		Civilians	
Group	SM	MFF N = 10	MPF N = 8	CM N = 8	CW N = 12
Height (cm)	P	178±4.1*^†^ (171–184)	182±7.0^†^ (173–193)	182±5.1^†^ (173–189)	170±7.5* (159–187)
VO_2max_ (L·min^−1^)	P	4.6±0.3^†^ (4.1–5.2)	4.4±0.4^†^ (3.9–5.3)	4.4±0.4^†^ (3.9–5.5)	3.2±0.6* (2.5–4.6)
VO_2max_ (mL·min^−1^·kg^·−1^)	P	58±4.4^†^ (52–65)	55±5.9*^†^ (43–61)	53±5.1*^†^ (48–64)^†^	47±7.0* (38–60)
30 m crawling (s)	P	13±2.4 ^†^ (10–18)	14±1.1^†^ (12–15)	15±3.7*^†^ (9–21)	19±3.5* (13–24)
3000 m running a (s·kg^−1^)	NP	9.5±2.1^†^ (8.1–11.0)	10.3±1.7*^†^ (7.6–11.7)	10.0±3.6*^ †^ (8.2–11.8)	12.0±6.2* (9.7–20.5)
Step test (% HR_max_)	P	76±7.3 ^†^ (59–85)	79±4.3^†^ (74–86)	84±4.9*^†^ (77–90)	89±5.9* (79–97)
Treadmill walking (% HR_max_)	P	76±5.4^†^ (62–83)	79±7.3*^†^ (69–89)	83±5.3*^†^ (73–90)	88±7.5* (75–100)
500 m rowing (s)	P	94±4.7 ^†^ (89–105)	96±5.2^†^ (89–106)	98±6.7^†^ (91–111)	115±39.0* (105–124)
500 m rowing (W)	P	430±58.0^†^ (306–503)	401±55.0^†^ (294–462)	378±70.2^†^ (258–473)	233±6.1* (190–298)
Stairs a, b (s)	P	65±8.2^†^ (51–82)	79±14.6^†^ (61–106)	78±19.4^†^ (53–111)	188±89.9* (95–374)
Pulling a (s)	P	15±2.5^†^ (11–19)	14±2.1^†^ (11–17)	19±3.5^†^ (15–24)	33±9.6* (19–49)
Demolition a (s)	P	53±11.8^†^ (30–72)	47±7.7^†^ (37–58)	45±8.8^†^ (36–58)	20±13.4* (1–44)
Rescue a (s)	P	19±3.5^†^ (16–25)	19±3.2^†^ (14–24)	22±5.1^†^ (17–31)	32±6.0* (23–40)
Terrain (s)	NP	645±103.0^†^ (528–716)	674±59.0^†^ (630–915)	683±63.0 ^†^ (609–786)	885±217.0* (693–1074)

Subject groups were: Male full-time firefighters (MFF), Male part-time firefighters (MPF), civilian men (CM), and civilian women (CW). Statistical Method (SM): Non-parametric tests (NP) are presented as medians ± Interquartile range (min-max), and parametric tests (P) are presented as means ± Standard deviation (min-max). One-Way ANOVA and Mann-Whitney (with Bonferroni correction) analysed subject group differences for P and NP variables, respectively. Only data for which there were significant differences among subject groups (p<0.01) are presented in the table, and marked with symbols in rows (*^, †^). Groups denoted with different symbols are significantly different (*different from^†^).

Investigated work tasks were: Carrying hose baskets up stairs (Stairs), Hose pulling (Pulling), Demolition at or after a fire (Demolition), Victim rescue (Rescue) and Carrying hose baskets over terrain (Terrain) Percentage use of maximal heart rate: % HR_max_. Performance in the 3000 m running test is presented as relative performance (in relation to body-weight: s·kg^−1^).

a *One CW subject did not begin the test.*

b *One CW subject did not complete the test.*

Firefighters were recruited from the Fire and Rescue Services in northern Sweden and civilians were recruited by notices at Luleå University of Technology and local gyms. All participants signed an informed consent stating their ability to execute all parts of the study and that they were free of any self-reported diseases or illnesses that could affect physical performance.

### Ethics Statement

The Research Ethics Committee for Northern Sweden at Umeå University approved the study on 22 September 2009 (Dnr 09–046M).

### Study Design

A previous study [26] and (Lindberg et al., unpublished) established the most common and physically demanding work tasks among Swedish firefighters. These tasks include *cutting holes in the roof for fire gas ventilation (Cutting)*, *carrying hose baskets in a staircase (Stairs)*, *hose pulling (Pulling)*, *demolition at or after a fire (Demolition)*, *victim rescue (Rescue), vehicle extrication (Vehicle)*, and *carrying hose baskets over terrain (Terrain).* To select and standardize physical tests of work performance, two laboratory aerobic capacity tests, six field tests, and the fore mentioned work tasks were performed. The tests were executed over 10 non-consecutive randomized days with each test day being separated by at least one non-testing day. Tests of muscle force and balance were also included in these 10 test days, but due to the extensive amount of data these results will be published separately. For all tests subjects wore shorts/pants, a t-shirt, and training shoes. Additional clothing and equipment that was worn or used in the tests is described below.

### Physical Tests

#### Aerobic capacity tests

On the first test day, submaximal treadmill running was performed and VO_2max_ was measured after a 10 min rest. Both oral and written instructions regarding diet and exercise prior to the tests were given in order to standardize each subject’s preparation.

#### Submaximal treadmill running

Subjects filled in a health questionnaire, and after 10 min of rest their arterial blood pressure was measured with a TriCUFF® (AJ Medical, Stockholm, Sweden), fingertip blood samples were taken for measurement of lactate concentration ([La^−^]_b_) (Biosen 5130; EKF-diagnostic, GmbH, Barleben, Germany), and mean B-Hemoglobin (B-Hb, Hemocue AB, Ängelholm, Sweden) from duplicate samples was recorded. Body weight and standing height were measured with a scale (SECA 770) and a stadiometer (Seca Corporation, Hanover, USA) wearing only shorts and a t-shirt. Before the test, the subjects warmed up for 10 min at a self-selected treadmill running speed (RL 1700 treadmill; Rodby Innovation AB, Södertälje, Sweden). Each subject performed 3–7 intervals of 4 min at a 0° incline, and speed was increased 1 km·h^−1^ for each interval until the Borg’s Ratings of Perceived Exertion (RPE) for chest (RPE_chest_) and legs (RPE_legs_) [27] reached 16–17 [28], [29]. Lactate threshold (LT) and Onset of blood lactate accumulation (OBLA have been suggested to occur at RPE 10–12 and around 16, respectively [29], [30]. Thus, RPE was used as an indicator that the subject had reached both LT and OBLA. LT and OBLA were determined by fingertip blood sampling at the end of each interval, and analyzed after the test was completed. LT measures the highest VO_2_ or exercise intensity that can be achieved without increasing [La^−^]_b_ by more than 1.0 mM [31], [32], and OBLA occurs at 4 mM blood lactate concentration [32], [33]. Running speed was individualized and started approximately 2 km·h^−1^ below the self-rated race-pace for 10 km running. The running speed ranged from 6–10 km·h^−1^ at the start with a mean of 8.1 km·h^−1^. Continuous measurements of heart rate (HR) (Polar heart rate monitor S810; Polar Electro Oy, Kempele, Finland), oxygen consumption (VO_2_), and respiratory exchange ratio (RER) were made (Jaeger Oxycon Pro; Erich Jaeger GmbH, Hoechberg, Germany, with Hans Rudolph accessories; Hans Rudolph Inc., Kansas city, USA). The mean values for HR, VO_2_, and RER were calculated during the final 60 s of each interval.

#### VO_2max_

The VO_2max_ test was performed at a fixed speed (the estimated maximal speed maintainable for 10 km). The treadmill incline increased by 1° each minute for the first 3 minutes, after which the incline increased by 0.5° every minute. Fingertip blood was sampled at 1 and 3 min after exhaustion, for measurement of maximal lactate concentration ([La^−^]_b max_). The 30 s recordings giving the highest mean for HR, VO_2_, and RER were considered the maximums during the test.

#### Field tests

A 6 min cycling test was performed on the second test day, and a 30 m crawling (Crawl) test was performed on the fourth test day. A 3000 m track running test was performed on the fifth test day and a step test was performed on the sixth test day. Treadmill walking for 6 min was performed on the seventh test day, and a 500 m rowing test was performed on the eighth test day.

#### Cycling

A cycling test (Ergomedic, 839 E; Monark Exercise AB, Vansbro, Sweden), used as a physical work capacity test for Swedish firefighters, was performed on the second day of testing [25]. After a 5 min warm-up at 50 W, the subjects cycled for 6 min at 200 W and 60 rpm (Korg MA-30 metronome, Korg and Moore, Marburg, Germany). Steady-state HR was calculated as the mean of test-minutes five and six, and the percent of maximal heart rate (% HR_max_) was calculated.

#### Crawl

A 30 m crawl test was performed on a flat plastic floor. The subjects wore kneepads and the test started with the subjects on their hands and knees. The subjects were instructed to crawl as fast as possible in the four-legged position and the time was stopped when their head crossed the finish line.

#### Track running

A 3000 m running test was performed on a 370 m indoor track after a 10 min self-selected warm up pace. The subjects were instructed to complete the test as fast as possible. Time and HR was recorded and % HR_max_ was calculated from the mean HR during the test. Both absolute time (s) and relative performance (s·kg^−1^) were recorded.

#### Step-test

Subjects performed a 6 min test consisting of 30 full steps·min^−1^ on a 20 cm high box. The subjects were dressed in personal protective gear including BA (the total weight of clothing and equipment was 24±0.5 kg). Steady-state HR was calculated as the mean of test-minutes five and six and the % HR_max_ was calculated. RPE_chest_ and RPE_legs_ [27] were rated at each minute, and the result from the final minute of the test was recorded.

#### Treadmill walking

A 6 min walking test, at 4.5 km·h^−1^ and an 8° incline, was performed according to the Work Health and Safety Agency’s standard [25]. The subjects were dressed with personal protective gear including BA. Steady-state HR was calculated as the mean of test-minutes five and six and the % HR_max_ was calculated.

#### Rowing

After a warm up consisting of 5 min of cycling at 50 W and 5 min rowing at a self-selected load, a 500 m rowing test was performed on a Concept II rowing machine (Concept Träningsredskap AB, Jönköping, Sweden) using the highest resistance (machine setting at 10) and with an anti-slip mat placed on the seat cushion. The subjects were instructed to complete the test as fast as possible starting when they chose to. The time (s) to complete the test and the mean power (W) generated were recorded using the built in software.

### Simulated Work Tasks

All work tasks below were performed at maximal speed and/or force.

**Figure 1:**
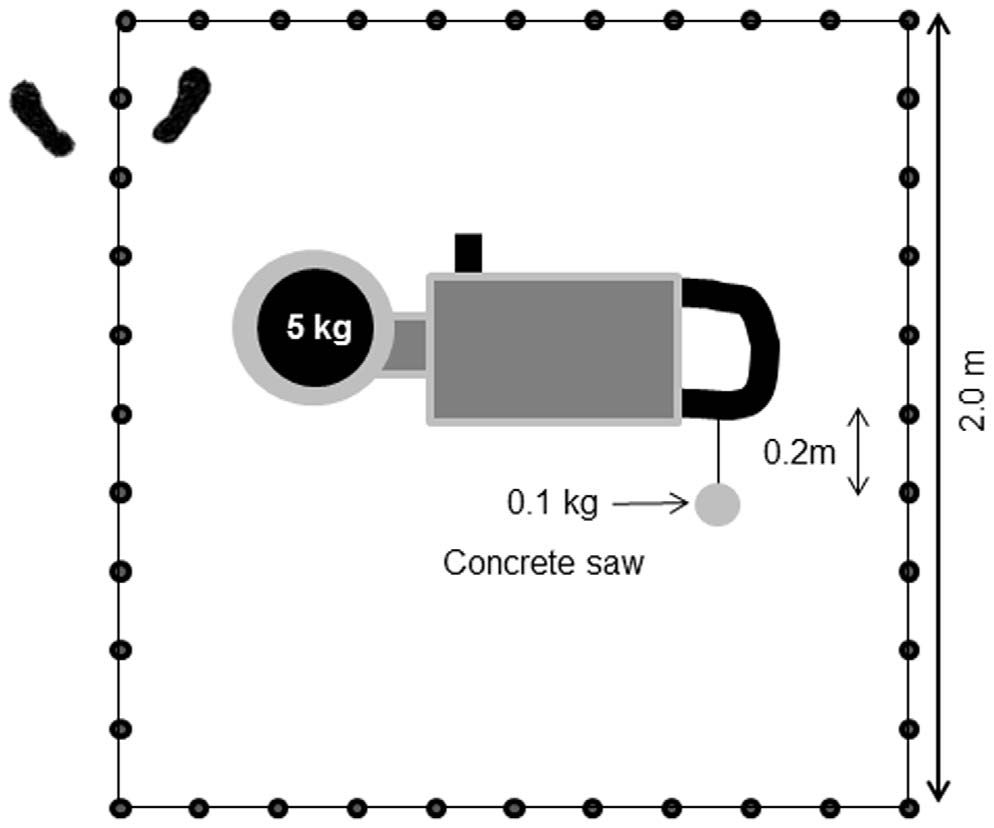
Schematic view of the*cutting holes in the roof for fire gas ventilation* work task. Subjects moved backwards along the markers, holding the concrete saw.

**Figure 2:**
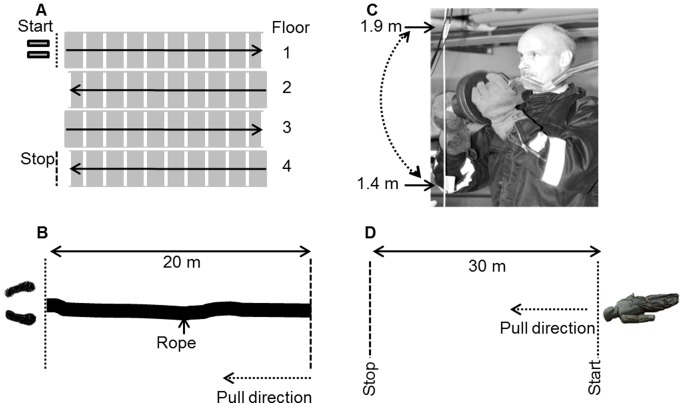
Schematic view of the work task course. The four work tasks: *carrying hose baskets in a staircase* (A), *hose pulling* (B), *demolition at or after a fire* (C) and *victim rescue* (D), was performed in sequence with two minutes of rest between each work task. The subject on the photograph has given written informed consent, as outlined in the PLOS consent form, to publication of their photograph.

**Figure 3:**
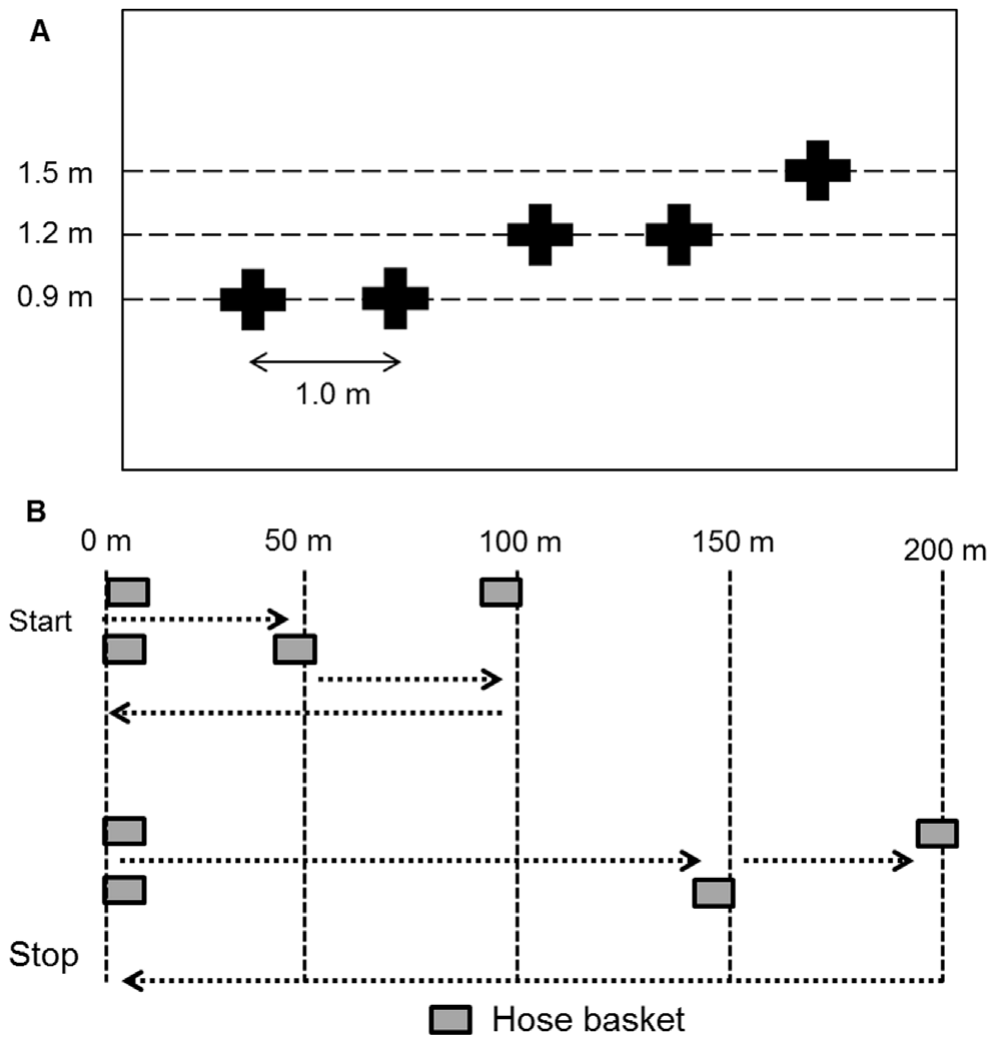
Schematic view of the*vehicle extrication* and *carrying hose basket over terrain* tasks. The work tasks were separated by 10 minutes of rest.

On the ninth test day, the *Cutting* task was performed (Figure 1). After 10 min of rest, a work task course including the *Stairs, Pulling, Demolition* and *Rescue* tasks (Figure 2) were performed in sequence with two minutes of rest between each work task. On the tenth test day the *Vehicle* and *Terrain* work tasks (Figure 3) were performed, separated by 10 min of rest. For all work tasks except the *Cutting* and *Vehicle* tasks, the HR was recorded and % HR_max_ was calculated. The times to complete all work tasks were recorded.

#### Cutting (Figure 1)

Marks were made every 0.2 m along a 2 m×2 m square drawn on the floor. An 11.0 kg concrete saw (Husqvarna 371 k, St. Olathe, USA) with a 5 kg weight taped to the blade and with a 0.1 kg weight attached to a 0.2 m string on the rear handle was used. The saw was placed in one corner and the subject’s feet were placed on either side of the line with one hand placed on each handlebar of the saw. At the start, the front part of the saw was raised 0.05 m above the floor and the rear 0.1 kg mass was kept in contact with the floor at all times. At a rate of 40 moves·min^−1^, the subjects moved backwards along the marks until voluntary exhaustion. The maximum time for the test was 15 min but this was not known by the subjects prior to the test. Supporting the arms on the legs was not allowed during the test.

During the *Stairs*, *Pulling*, *Demolition*, and *Rescue* work tasks (Figure 2), the subjects were dressed in a fire emergency jacket, gloves, and BA (19±0.5 kg).

#### Stairs (Figure 2A)

Two hose baskets (each basket was designed for two 25 m long, 42 mm diameter hoses, and the weights of the baskets were adjusted to 16.0 kg) were carried up 4 floors (step height 0.17 m, width 0.19 m, and a total vertical rise of 13 m) two times, with a 60 s rest period while walking down. The subjects were instructed to complete the test as fast as possible. Performance was registered as the total time to complete the two laps excluding the rest period.

#### Pulling (Figure 2B)

A 25 m long, 70 mm diameter rope was pulled 20 m as fast as possible using only the arms and without moving the feet. Pull resistance at full-length was determined to be approximately 220 N by slowly pulling the rope on a cement floor at constant speed with a force dynamometer (Grip-D; Eleiko Sport AB, Halmstad, Sweden).

#### Demolition (Figure 2C)

A 1.16 m Z-bar (8.5 kg) (Casall Sports Products AB, Norrköping, Sweden) was used with one end loaded with 3×2.5 kg weights and a 0.25 kg lock placed at both ends of the bar. The end of the bar not loaded with weights was attached to the celling. The attachment point at the Z-bar was 1.90 m above the floor. A string was attached between the floor and the ceiling and marked at 1.40 m and 1.90 m above the floor making the lifting momentum for the tested subject approximately 137 Nm and the range of movement 0.5 m. The Z-bar was lifted between the 1.40 m and 1.90 m marks with a frequency of 25 lifts·min^−1^ until voluntary exhaustion.

#### Rescue (Figure 2D)

A 75 kg rescue doll was pulled across a concrete floor for 30 m using a chest harness. The subjects were instructed to grip the chest harness placed around the upper body of the doll before starting the test. At the start signal, the subjects moved the doll as fast as possible backwards. Time stopped when the head of the doll crossed the finish line.

#### Vehicle (Figure 3A)

Five points at three different heights (0.9, 1.2, and 1.5 m) from the floor were marked on a wall. An 18.5 kg spreader (Holmatro SP 3240t; Wennergren Maskin AB, Grimslöv, Sweden) was held with both hands. The front part was pressed against each point for 15 s, and then moved to the next point in the following pattern: 0.9–0.9–1.2–1.2–1.5–1.2–1.2–0.9–0.9 m. The angle of the spreader was always 45° from the body and the spreader was not allowed to be placed on the shoulder or on the hip. The test was performed to voluntary exhaustion, but with a maximum time of 10 min (not known to the subjects before the test).

#### Terrain (Figure 3B)

Two baskets (each basket designed for two 25 m long, 63 mm diameter hoses, adjusted to 18.7 kg) were carried 50 m. One basket was dropped, and the other basket was carried another 50 m. The subject then moved 100 m without baskets. Two more baskets were then carried 150 m, one was dropped, and the other carried another 50 m. The subjects then moved 200 m without baskets. The course was repeated for three laps, but the last 200 m without baskets was excluded on the third lap and resembled a real time situation in which the next work task would start. A total movement of 1600 m (900 m with baskets and 700 m without baskets) was performed on the concrete floor with subjects wearing gloves. The subjects were instructed to complete the course as fast as possible.

### Statistics

Statistical calculations were carried out with SPSS version 20.0 (IBM Corporation, USA). Parametric variables are presented as means ± SD (min-max) and non-parametric variables are presented as median ± Interquartile range (IQR) (min-max) [34]. Data was assumed to be normally distributed if two out of three parameters were achieved: skewness and kurtosis ranged within ±2.58 of standard error, the Shapiro-Wilk’s test was >0.05 and the Q–Q Plot was approximately normally distributed, visually inspected [35]. Comparisons between subject groups were assessed using either one-way ANOVA with post hoc Bonferroni correction (for non-skewed, parametric variables), or Kruskal-Wallis and Mann Whitney tests for non-parametric and skewed variables. When significant differences were found with the Kruskal-Wallis test, the Mann-Whitney U-test was carried out, using post hoc Bonferroni correction to avoid a Type 1 error: the p-value was divided with the number of paired comparison.

Spearman’s rank correlation coefficient (r_s_) was used to analyze correlations between dependent and independent variables. A p-value <0.01 was considered statistically significant for all tests.

## Results

### Physical Tests Performances

#### Aerobic capacity tests

OBLA was reached at a mean treadmill speed of 11.6±1.9 (8.5–15.5) km·h^−1^, 88±4.7 (78–97) % HR_max_, 81±5.0 (68–90) % VO_2max_ and median RPE_chest_ 14±3.0 (9–17)_ and_ RPE_legs_ 13.8±2.5 (10–17). LT was reached at a mean treadmill speed of 11.0±2.0 (6–15) km·h^−1^, 86±4.5 (76–92) % HR_max_ and 77±4.1 (69–85) % VO_2max_ and median RPE_chest_ 13±4 (7–17) and RPE_legs_ 13±4 (6–17). No significant differences were observed between groups. Four subjects (2 MFF, 1 MPF, and 1 CW) did not reach OBLA, and five subjects (2 MFF, 1 MPF, 1 CM, and 1 CW) did not reach LT.

The mean time to exhaustion on theVO_2max_ was 342±74 (226–540) s with a fixed mean treadmill speed of 12.5±1.5 (9–15) km·h^−1^ and a final incline of 3.9±0.7 (2.5–5.5) °. The mean treadmill speed was higher for MFF and MPF compared to CW. CW reached lower VO_2max_ (L·min^−1^) compared to all groups of men, and lower VO_2max_ (mL· kg^−1^· min^−1^) compared to MFF (Table 1). There were no differences in the mean [La^−^]_bmax_ (12±2.8 (7–21) mmol·L^−1^) or RER_max_ (1.1±0.06 (0.94–1.3)) between groups, and 76% and 95% of the subjects reached RER >1.1 and >1.0, respectively.

#### Field tests

Five CW were unable to complete the 6 min cycling, stopping at a mean time of 2 min 54 s ±44 s (2 min 0 s–3 min 53 s) and 94±2.7 (91–97) % HR_max_. For the subjects completing the test, no differences were found between the groups’ mean % HR_max_ (79±8.8 (58–92) %).

Completion time for the 30 m crawl test was faster for MFF and MPF compared to CW (Table 1).

The mean time on the 3000 m track running test was 842±1325 (642–1325) s, and the mean HR averaged 93±3.0 (87–99)% of HR_max_ during the test. No significant differences between subject groups were observed. When running time was related to body weight (s·kg^−1^), CW had lower performance compared to MFF (Table 1). One CW subject did not begin the test.

The step test was performed at a higher % HR_max_ at steady-state for CW compared to MFF and MPF (Table 1). Median RPE_chest_ and RPE_legs_ (15±3 (7–18) and 15±3 (7–19), respectively) did not differ between groups.

CW had higher steady-state % HR_max_ compared to MFF during the 6 minute treadmill walking test (Table 1).

All groups of men completed the 500 m rowing test faster, and at a higher mean power, than CW (Table 1).

#### Simulated work tasks

Due to the large number of subjects reaching maximal time (n = 33 (87%)), the *Vehicle* task was removed from further data analysis. The mean performance time in the *Cutting* tasks was without significant differences between subject groups: 322±179 (115–900) s, one CW subject did not perform the test. All men reached higher performance compared to CW in the *Stairs*, *Pulling*, *Demolition* and *Rescue* work tasks (Table 1). The *Terrain* task was executed faster by MFF and CM compared to CW (Table 1). No differences were found between groups in mean % HR_max_ for the work task course or the *Terrain* work task (84% ±4.5 (75–91) and 89% ±4.7 (78–96), respectively). One CW subject did not perform the work task course, and one CW subject was not able to complete the *Stairs* work task.

### Correlations

#### Correlations between aerobic capacity tests and simulated work tasks

Performance time in the *Terrain* task was the only work task significantly correlated with treadmill speed at OBLA (r_s_ = −0.65) and LT (r_s_ = −0.63) (Table 2). Work tasks performance times were not significantly correlated with % HR_max_ at OBLA and LT or with % VO_2max_ at OBLA and LT (Table 2). The average % HR_max_ during the work task course (*Stairs, Pulling, Demolition* and *Rescue*) and the *Terrain* task were without significant correlations with %VO_2max_ at OBLA (r_s_ = −0.17 and 0.27, respectively) and LT (r_s_ = −0.13 and 0.12, respectively), and also without correlations to % HR_max_ at OBLA (r_s_ = −0.07 and 0.35, respectively), and LT (r_s_ = −0.02 and 0.32, respectively).

**Table 2:** Correlations between performance in physical tests and simulated work tasks.

		Cutting (s)	Stairs (s)	Pulling (s)	Demolition (s)	Rescue (s)	Terrain (s)
	N	37	36	37	37	37	38
OBLA speed (km·h^−1^)	34	0.41	−0.24	−0.34	0.37	−0.31	−0.65*
OBLA % HR_max_	34	−0–15	−0.23	0.29	−0.18	0.26	−0.18
OBLA % VO_2max_	32	−0.03	−0.09	0.02	−0.08	−0.03	−0.22
LT speed (km·h^−1^)	33	0.42	−0.25	−0.38	0.41	−0.35	−0.63*
LT % HR_max_	32	−0.12	−0.18	0.32	−0.26	0.23	−0.30
LT % VO_2max_	33	−0.04	−0.07	−0.06	−0.05	−0.01	−0.09
VO_2max_ (L·min^−1^)	38	0.55*	−0.75*	−0.74*	0.79*	−0.79*	−0.70*
VO_2max_ (mL·min^−1^·kg^·−1^)	38	0.47*	−0.52*	−0.46*	0.57*	0.48*	−0.74*
Cycling (% HR_max_)	33	−0.56*	0.68*	0.69*	−0.74*	0.66*	0.63*
30 m crawling (s)	38	0.49*	0.74*	0.62*	−0.57*	0.70*	0.41
3000 m running (s)	37	−0.45*	0.36	0.41	−0.53*	0.41	0.67*
3000 m running (s·kg^−1^)	37	−0.54*	0.59*	0.72*	−0.78*	0.68*	0.69*
Step test (% HR_max_)	38	−0.38	0.58*	0.66*	−0.69*	−0.54*	0.69*
Treadmill walking (% HR_max_)	38	−0.61*	0.54*	0.57*	−0.59*	0.48*	0.71*
500 m rowing (s)	38	−0.63*	0.82*	0.76*	−0.70*	0.79*	0.65*
500 m rowing (W)	38	0.63*	−0.83*	−0.75*	0.70*	−0.79*	−0.65*

Correlations were analysed with Spearman’s rank correlation coefficients. Investigated work tasks were: *Cutting holes in the roof for fire gas ventilation* (Cutting), *Carrying hose baskets in a staircase* (Stairs), *Hose pulling* (Pulling), *Demolition at or after a fire* (Demolition), *Victim rescue* (Rescue) and Ca*rrying hose baskets over terrain* (Terrain).

OBLA: Onset of blood lactate accumulation, LT: Lactate threshold. Percentage of maximal heart rate: % HR_max_. Performance in the 3000 m running test is presented as absolute (s) and relative performance in relation to body-weight (s·kg^−1^).

* *p<0.01 N = Number of subjects completing the work task and the physical test.*

The performances times for five of the six work tasks had a higher correlation with VO_2max_ in L·min^−1^ than mL·kg^−1^·min^−1^. Only the performance time for the *Terrain* task had a higher correlation to VO_2max_ in mL·kg^−1^·min^−1^ (Table 2).

#### Correlations between aerobic capacity tests and field tests

Treadmill speed at OBLA and LT was significantly correlated to performance in all field tests except % HR_max_ during the cycling, crawling, and rowing tests. The highest correlation to OBLA and LT was observed for running time on 3000 m (s) (r_s_ = −0.84 and −0.85, respectively) (Table 3).

**Table 3:** Correlations between aerobic capacity tests and field tests.

	OBLA speed (km·h^−1^)	LT speed (km·h^−1^)	VO_2max_ (L·min^−1^)	VO_2max_ (ml·min^−1^·kg^·−1^)	Cycling (% HR_max_)	30 m crawling (s)	3000 m running (s)	3000 m running (s·kg^−1^)	Step test (% HR_max_)	Treadmill walking (% HR_max_)	500 m rowing (s)
OBLA speed (km·h^−1^)	1	0.95*	0.43	0.72*	−0.21	−0.23	−0.84*	−0.51*	−0.70*	−0.65*	−0.34
LT speed (km·h^−1^)		1	0.45*	0.71*	−0.24	−0.22	−0.85*	−0.53*	−0.66*	−0.65*	−0.35
VO_2max_ (L·min^−1^)			1	0.65*	−0.72*	−0.60	−0.52*	−0.85*	-−0.66*	−0.65*	−0.84*
VO_2max_ (mL·min^−1^·kg^·−1^)				1	−0.29	−0.50*	−0.84*	−0.50*	−0.69*	−0.74*	−0.51*
Cycling (% HR_max_)					1	0.36	0.64	0.32	0.68*	0.72*	0.62*
30 m crawling (s)						1	0.35	0.49*	0.50*	0.37	0.69*
3000 m running (s)							1	0.66*	0.67*	0.69*	0.40
3000 m running (s·kg^−1^)								1	0.64*	0.61*	0.73*
Step test (% HR_max_)									1	0.81*	0.63*
Treadmill walking (% HR_max_)										1	0.60*
500 m rowing (s)											1

* p<0.01. OBLA: Onset of blood lactate accumulation, LT: Lactate threshold. Percentage of maximal heart rate: % HR_max_. Performance in the 3000 m running test is presented as absolute (s) and relative performance in relation to body-weight (s·kg^−1^).VO_2max_: maximal oxygen uptake* p<0.01.

All field tests were significantly correlated with both VO_2max_ (L·min^−1^) and VO_2max_ (mL·kg^−1^·min^−1^). The highest correlation to VO_2max_ (L·min^−1^) was found in relative performance on the 3000 m track running test (s·kg^−1^), and the highest correlation to VO_2max_ (mL·kg^−1^·min^−1^) was found in absolute performance in seconds for the 3000 m track running test (Table 3).

#### Correlations between field tests and simulated work tasks

Performances in the field tests were significantly correlated to performance in at least three work tasks (Table 2). Performance in the rowing (s), track running (s·kg^−1^), and treadmill walking (% HR_max_) tests had the highest correlations (highest r_s_ values) to work task performance.

## Discussion

We and others have shown that aerobic capacity is important for firefighters work performance [5], [12], [18], [19], [20], [21], [22], [24], [36], [37]. The main finding in this study is that there are strong correlations between direct (laboratory) and indirect (field) aerobic capacity tests and commonly occurring, and physically demanding firefighting tasks. Also, indirect (field) tests may serve equally well as more advanced (direct) aerobic capacity tests for prediction of firefighters work performance.

### Aerobic Capacity and Work Performance

As a group, and as expected [19], [20], [38], women performed more poorly on the simulated work tasks than men. However, on all tests some women performed better than some men indicating that none of the included simulated work tasks were necessarily discriminative based on gender. Performance in five of the six work tasks had higher correlation to VO_2max_ in L·min^−1^ compared to VO_2max_ in mL·kg^−1^·min^−1^. This is in contrast to the findings by Harvey et al. [39] that showed low correlations between VO_2max_ (L·min^−1^ and mL·kg^−1^·min^−1^) and completion time on a work-task circuit, but is in accordance with other studies [12], [21], [22], [36]. Other studies have used only VO_2max_ measured in mL·kg^−1^·min^−1^ as a standard when measuring firefighter work performance [18], [20], [24], but the results of the present study suggest that it is more relevant to use VO_2max_ measured in in L·min^−1^ for the purpose of evaluating firefighters’ aerobic work capacity.

### Aerobic Capacity and Field Tests

As a group, and as expected [40], [41], women reached lower VO_2max_ (L·min^−1^), had higher physical strain, and performed more poorly than men in several of the investigated field tests. However, on all tests some women performed better than some men indicating that none of the included tests are discriminative based on gender. This is important in the context of both test and personnel selection.

Direct measurements of VO_2_, LT and OBLA are complicated, time consuming, and expensive. Thus, substitute performance tests that correlate to LT, OBLA, VO_2max_ or to work performance are preferred in the selection and evaluation process of personnel.

A higher (negative) correlation between VO_2max_ (L·min^−1^) and performance in the 3000 m running test was observed when the performance was expressed relative to body weight (s·kg^−1^; r_s_ = −0.85) than when performance was expressed only in absolute time (s; r_s_ = −0.52). VO_2max_ measured in L·min^−1^ was correlated to both time and mean generated power in the 500 m rowing test (r_s_ = −0.84 and r_s = _0.84, respectively). These three variables could be used for estimation of a firefighter’s work performance with the understanding that other qualities, such as muscle strength, muscle endurance, and anaerobic capacity [3], [21], [22], [23], [24], [38], [42], [43], can also affect the work performance.

### Field Tests and Work Performance

Tests that are simple to administer, yet reliable and having high validity, are important when selecting personnel for physically demanding work. Valid measurements of aerobic capacity are difficult to achieve in a work place setting. Some of the present field tests were selected because similar tests have been included in published studies [10], [11] or are used as standard medical tests in the Swedish fire and rescue services [25]. Others were designed based on common exercise science assumptions. For example, the 3000 m running, 500 m rowing, and 30 m crawling tests have not been included in any published study investigating firefighter work performance. The most commonly used tests are determination [8], [11], [18], [19], [20], [21], [22], [24], [36], [39], [43] or prediction of VO_2max_ using other measures such as: submaximal treadmill running [15] or submaximal step test [10], [44].

Significant correlations ranging from 0.48 to 0.71 (in absolute r_s_) were found between the % HR_max_ during the treadmill walking test and the investigated work tasks. The practical use of such tests, however, is problematic because the subjects’ maximal heart rates (HR_max_) are usually not known.

### Limitations

There are very few female firefighters, and none could be recruited for this study. The lack of participating females resulted in unknown performance variables for female firefighters and prevented accurate comparison to males. Most studies investigating correlations between results on physical performance tests and firefighting work tasks include male subjects only [18], [21], [23], [36], [42], [45], [46] or merge results from men and women [20], [22], [38]. Harvey et al. [39] and Williams-Bell et al. [24] found different correlations between simulated work tasks and field tests for men and for women, but they merged the groups in the multivariate analyses. Consequently, no published study has determined if there are different limiting factors for men and women in firefighting work performance. By using larger subject groups, and including more women in future studies, this can be investigated.

All subjects did not perform all tests. The largest loss of data was found in the cycling test, and in measurements of OBLA and LT. Five subjects did not complete the cycling test. All but one of these subjects had a VO_2max_ lower than 2.8 L·min^−1^, and the required VO_2_ for cycling at 200 W is approximately 2.8 L·min^−1^ [47]. Analysis of [La^−^]_b_ was performed after the completion of the treadmill running test, adjustments in speed during the test in order to reach OBLA and LT were not made and four and five values are, therefore, missing from the final analysis for OBLA and LT, respectively.

### Conclusion

Because of the significant correlation between test results and work task performance, our results suggest that aerobic capacity is important for performance on commonly occurring, and physically demanding, firefighting work tasks. Results on both direct (laboratory) and indirect (field) tests are correlated to work task performance.

Recommended field tests for evaluation of firefighters’ work performance are: time on 500 m rowing (s), 3000 m running relative to body weight (s·kg^−1^), and the percent of maximal heart rate achieved during 6 min treadmill walking at 4.5 km·h^−1^ and 8° incline. Future studies should investigate limiting factors for firefighting work performance, and if these limits differ between men and women.
